# Prenatal care and socioeconomic status: effect on cesarean delivery

**DOI:** 10.1186/s13561-018-0190-x

**Published:** 2018-03-10

**Authors:** Carine Milcent, Saad Zbiri

**Affiliations:** 1 Paris-Jourdan Sciences Economiques, French National Center for Scientific Research, Paris, France; 2 EA 7285, Versailles Saint Quentin University, Montigny-le-Bretonneux, France

**Keywords:** Cesarean delivery, Pregnancy care, Health education, Socioeconomic position, I12, I14, I18

## Abstract

Cesarean deliveries are widely used in many high- and middle-income countries. This overuse both increases costs and lowers quality of care and is thus a major concern in the healthcare industry. The study first examines the impact of prenatal care utilization on cesarean delivery rates. It then determines whether socioeconomic status affects the use of prenatal care and thereby influences the cesarean delivery decision. Using exclusive French delivery data over the 2008–2014 period, with multilevel logit models, and controlling for relevant patient and hospital characteristics, we show that women who do not participate in prenatal education have an increased probability of a cesarean delivery compared to those who do. The study further indicates that attendance at prenatal education varies according to socioeconomic status. Low socioeconomic women are more likely to have cesarean deliveries and less likely to participate in prenatal education. This result emphasizes the importance of focusing on pregnancy health education, particularly for low-income women, as a potential way to limit unnecessary cesarean deliveries. Future studies would ideally investigate the effect of interventions promoting such as care participation on cesarean delivery rates.

## Background

Health expenditures are high and continue to rise in many parts of the world, despite the financial and economic crisis that slowed the trend in some countries. This spending grew during the 2009–2016 period by 5.7% in South Korea, 2.8% in Switzerland, 2.7% in Australia, 2.1% in the United States, 1.8% in Germany and Japan, 1.1% in Canada, 0.9% in France and the United Kingdom [1]. There is therefore considerable pressure to rein in health costs. Unnecessary medical practices are usually pointed out as a cause of escalating healthcare spending [2]. Previous literature has looked into the ballooning overuse of medical services and describes it, to a large extent, as a widespread phenomenon that is difficult to address and that remains understudied [3–5]. In this research, we focus on cesareans, a mode of delivery generally considered to be overused in many industrialized countries [6, 7], and explore the associations between cesarean delivery use, prenatal care utilization, and socioeconomic status.

Cesarean deliveries are currently one of the most common surgical procedures in the world; approximately 18 million are performed yearly worldwide, almost 6 million more than the 10–15% rate recommended by World Health Organization (WHO) [8]. Indeed, since 1985, WHO has stated that the number of cesareans should range between 10 and 15% of total deliveries, regardless of the region or the country [9]. This recommendation was reaffirmed in 2015 [10]. These presumptively unnecessary cesarean deliveries are responsible for an additional cost of more than 2 billion US dollars and for an unquantified burden on the health system through pressure on medical resources [8]. The surgical approach to delivery is associated with significantly greater economic costs than other modes of delivery. Xu et al. [11] find that estimated facility costs for low-risk childbirth are higher at hospitals with higher cesarean delivery rates. Similarly, Allen et al. [12] report that cesarean deliveries, especially those performed during labor, are associated with the highest hospital costs of delivery care. Moreover, when performed for first deliveries, they are associated with higher cumulative costs than other methods of delivery, regardless of the number or type of subsequent deliveries [13]. Evidence also suggests that attempted vaginal delivery (i.e., trial of labor) is a more cost-effective option for women with a previous cesarean than an elective repeat cesarean delivery, although its cost-effectiveness depends on the probability of successful vaginal delivery [14]. On the other hand, cesarean deliveries do not lead to better health outcomes for women or infants who do not require the procedure and can even cause short- and long-term adverse health effects [15–17].

The use of cesarean deliveries results from a wide array of medical and non-medical factors. Although specific medical risk factors are indications for cesarean delivery, they provide a very incomplete explanation of all cesarean deliveries [18]. Women’s characteristics and preferences influence their mode of delivery [19], as do physicians’ attitudes and incentives toward birth management [20, 21] and the financing and organizational structures of hospitals [22, 23].

Women’s socioeconomic status is one of the individual determinants of cesarean deliveries. Many results have showed differences in the mode of delivery across socioeconomic groups, often assessed by income, occupation, or education, even after adjustment for medical risk factors. Overall, since the 2000s, analyses from developed countries have found that women of lower socioeconomic position are more likely than their better-off counterparts to have cesarean deliveries. In France, Guihard and Blondel [24] report that women with a low level of education have a higher risk of cesarean deliveries. German studies point out high rates of surgical deliveries for low-income women [25, 26]. Accordingly, Italian mothers with low education levels consistently give birth by a cesarean delivery more often than highly educated women [27]. Similarly, Lee et al. [28] describe lower cesarean delivery rates in South Korea for women with higher education or occupational levels. In a population of US Defense Department personnel, higher cesarean delivery rates were significantly associated with lower socioeconomic status [29]. From 1967 to 2004, women with the lowest level of education in Norway had the highest probability of cesarean deliveries [30]. Finally, Joseph et al. [31] find that the most affluent Canadian women are less likely to have a cesarean delivery than those in the lowest income category. The existing literature explains the high rates of cesarean deliveries for low-income patients in several ways, including by a greater preference for cesarean deliveries and a low access to obstetric care [25, 30]. However, no consensus has been reached on the underlying reasons.

Prenatal care is an important preventive healthcare service throughout pregnancy. Besides routine health evaluations, it provides education and counseling, as well as any necessary treatment [32]. WHO [33] in its *Standards for Maternal and Neonatal Care* recommends that all pregnant women have at least four antenatal assessments, starting as early as possible in the first trimester, conducted under the supervision of a skilled attendant, and spaced at regular intervals. Previous studies have examined the potential effect of organized prenatal care on perinatal health and showed it to be effective in reducing maternal mortality and serious morbidity [34]. Yan [35] reports that poor prenatal care increases the risks of many maternal complications and poor health habits during pregnancy as well as after delivery. Specifically, the increased adverse health outcomes are: insufficient gestational weight gain, prenatal smoking, premature rupture of membranes, preterm labor, no breastfeeding, postnatal underweight, and postpartum smoking. Moreover, the receipt of prenatal care is linked to different positive neonatal outcomes, in particular, reduced rates of both preterm birth and low birth weight. Blondel and Marshall [36] in France and Krueger and Scholl [37] in the United States report a higher probability of preterm deliveries among women with poor attendance at prenatal visits. Rous et al. [38] find that each additional prenatal visit is associated with higher birth weight. More broadly, Partridge et al. [39] observe that the risk of prematurity, stillbirth, and neonatal or infant death increases linearly with decreasing prenatal care utilization. Accordingly, Raatikainen et al. [40] find significantly more low-birth-weight infants and more fetal and neonatal deaths among woman with inadequate prenatal care. Finally, beyond its clear medical benefits, prenatal care may also have a psychological effect, preparing women for childbirth and motherhood [41].

While much of the research attention has focused on the relation between pregnancy care and maternal morbidity and mortality, several other health outcomes may be modified by prenatal care and require further investigation. The effect of prenatal care on method of delivery has not yet been adequately investigated. The primary objective of our study is thus to investigate the impact of routine prenatal care on cesarean delivery rates while simultaneously verifying the influence of socioeconomic conditions on these rates. A complementary objective of the analysis is to identify the socioeconomic determinants of the use of any type of prenatal care that significantly affects mode of delivery.

Our paper proceeds as follows. After this brief introduction to the conceptual framework of the study and previous literature, we describe the data used and explain our econometric strategy. We then present the descriptive analysis, provide the empirical findings, and discuss the results. Finally, we draw a general conclusion from the study.

## Methods

### Data

We have access to data that allow us to determine: *i)* women’s use of prenatal care, *ii)* their socioeconomic position, and *iii)* many other patient and hospital characteristics. Specifically, our analysis uses unique French delivery data of all births recorded in the Yvelines administrative district for the period of 2008–2014. The dataset comes from the infant first health certificates (The *Premiers Certificats de Santé (PCS)*). Some supplementary information is secondarily extracted from an additional health certificate. This dataset contains patient demographic information; socioeconomic status of the household; detailed information about the pregnancy and delivery including prenatal care utilization, hospital stays, date and place of birth, type of delivery and procedures performed; and full information about maternal, fetal, and neonatal health. Because the certificates do not provide complete information about the hospital where the woman gave birth, we use a dataset of French annual hospital statistics (The *Statistique Annuelle des Etablissements de santé (SAE)*), a national survey conducted by the Ministry of Health and covers all French hospitals. Hence, we have information for each hospital in the Yvelines on its type, activity, organization, and medical staff configuration. Staffing is reported in terms of full-time equivalents (FTEs), except that private-practice physicians in private hospitals are reported only by number of individuals per hospital. We therefore apply a hypothetical average to take the level of work of these physicians working part-time in hospitals into account by considering that they devote 50% of their time to that practice. This method has been used by previous studies [42]. We also check that all our results are robust to the more extreme assumptions of 25% and 75%. Full tables of these results are not presented, in view of their similarity to those using the average assumption, but are available upon request.

Our analysis covers all live deliveries performed in the 11 hospitals of the Yvelines district from January 2008 to the end of December 2014. Thus, our full sample includes 102,236 observations. However, information on prenatal care use and socioeconomic status is missing for some women, thereby restricting the corresponding analyses to 68,314 and 58,324 observations, respectively. Because the two characteristics studied, prenatal care and socioeconomic status are generally highly correlated with the patient’s health status, we also identify a subsample of low-risk women. To study cesarean delivery use, our low-risk subsample comprises nulliparous women aged 20–34 years, without any diagnosis or co-morbidity, giving birth at full term, during labor, without induction, to a singleton infant in cephalic presentation, and with a normal birth weight (20,683 observations). Prenatal care participation is also studied separately in the same low-risk sample but which includes only vaginal deliveries (10,947 observations). The definition of the low-risk population is based on the medical criteria generally used [43, 44]. The quality of the data extracted is high: the medical information is completed by health professionals and data collection is regularly checked. To verify the reliability of its information, the dataset undergo a double quality control that enables the correction of false and missing data. In addition, hospital information extracted from the French annual statistics for hospitals is checked and supplemented with data from hospitals. Furthermore, the data are reported to the French data protection authority (The *Commission Nationale de l’Informatique et des Libertés (CNIL)*), de-identified, and routinely used for health statistics; accordingly, French law does not require specific written informed consent from patients. The summary statistics of the key variables of our analysis are reported in Tables 1 and 2. The mean cesarean delivery rate for the full sample is 23.9% over the study period. The French national rate was around 21% in 2010 [45].

**Table 1:** Cesarean delivery rates according to prenatal care utilization and household socioeconomic characteristics

	Full sample (*N* = 102,236)			Low-risk subsample (*N* = 20,683)		
	Deliveries, %	Cesarean rate, %	Chi-square test	Deliveries, %	Cesarean rate, %	Chi-square test
Prenatal care						
First antenatal visit	(*n* = 100,672)			(*n* = 20,357)		
First trimester	98.5	23.9	3.7	98.7	11.4	7.3^b^
Second trimester	1.2	23.0		1.0	14.7	
Third trimester	0.3	19.8		0.3	21.1	
Obstetric ultrasounds	(*n* = 94,686)			(*n* = 19,276)		
< 3	1.7	20.6	1000.0^a^	1.1	11.5	0.7
= 3	68.3	20.8		76.1	11.4	
≥ 4	30.0	30.4		22.8	11.9	
Nuchal translucency ultrasound	(*n* = 98,643)			(*n* = 19,972)		
Yes	97.1	23.9	12.5^a^	97.5	11.5	0.5
No	2.9	21.1		2.5	12.5	
Morphology ultrasound	(*n* = 98,334)			(*n* = 19,885)		
Yes	98.9	24.0	28.2^a^	99.1	11.5	0.8
No	1.1	17.0		0.9	9.3	
Early prenatal interview	(*n* = 88,690)			(*n* = 17,882)		
Yes	20.4	22.3	15.1^a^	27.9	10.6	2.6
No	79.6	23.7		72.1	11.4	
Prenatal education	(*n* = 82,417)			(*n* = 17,159)		
Yes	54.9	21.9	99.1^a^	75.8	10.9	15.6^a^
No	45.1	24.9		24.2	13.2	
Woman’s socioeconomic level						
Familial situation	(*n* = 100,411)			(*n* = 20,328)		
Married or cohabiting	98.0	23.8	1.4	97.8	11.6	1.1
Single	2.0	25.0		2.2	10.0	
Healthcare coverage	(*n* = 100,146)			(*n* = 20,253)		
Insured	98.5	23.9	1.3	98.5	11.6	0.8
Uninsured	1.5	22.7		1.5	9.9	
Education	(*n* = 79,428)			(*n* = 16,080)		
Primary school	2.7	24.9	62.1^a^	1.3	16.2	45.4^a^
Some secondary school	14.0	25.2		10.4	13.0	
Completed secondary school	21.9	25.4		21.8	13.3	
College or university	61.4	22.8		66.5	9.8	
Occupation	(*n* = 70,201)			(*n* = 14,818)		
Manual worker	1.4	26.7	45.6^a^	1.1	13.8	26.7^a^
Office, sales, or service staff	55.2	24.9		57.8	12.0	
Farmer	0.3	24.4		0.3	18.2	
Crafts/trades worker or entrepreneur	2.9	25.5		2.8	11.4	
Intermediate (technical)	9.2	23.4		9.6	10.8	
Managerial or higher intellectual	31.0	22.7		28.4	9.1	
Work status	(*n* = 83,351)			(*n* = 16,838)		
Working	69.6	24.0	22.4^a^	75.8	10.9	2.2
Unemployed	6.7	25.1		7.3	10.9	
Not in labor force	23.7	22.6		16.9	11.9	
Partner’s socioeconomic level						
Occupation	(*n* = 78,712)			(*n* = 15,826)		
Manual worker	9.3	24.5	19.0^a^	8.2	11.7	17.4^a^
Office, sales, or service staff	40.1	24.2		43.6	11.8	
Farmer	0.4	24.8		0.3	17.3	
Crafts/trades worker or entrepreneur	7.3	23.6		6.5	10.2	
Intermediate (technical)	6.2	23.2		6.6	8.5	
Manager or higher intellectual	36.7	22.8		34.8	10.2	
Work status	(*n* = 79,154)			(*n* = 16,148)		
Working	89.8	23.6	6.3^b^	89.8	10.9	2.2
Unemployed	4.4	24.7		4.1	12.8	
Not in labor force	5.8	24.9		6.1	10.8	

The full sample is composed of all live deliveries performed in the hospitals of the French administrative district of Yvelines in 2008–2014. The low-risk subsample only includes those deliveries of nulliparous women aged 20–34 years, without any diagnosis or co-morbidity, giving birth at full term, during labor, without induction, to a singleton infant in cephalic presentation, and with a normal birth weight

^a^ = 1% significance level, ^b^ = 5%

**Table 2:** Prenatal education attendance rates according to household socioeconomic characteristics

	Full sample (*N* = 82,417)			Low-risk subsample (*N* = 10,947)		
	Deliveries, %	Attendance rate, %	Chi-square test	Deliveries, %	Attendance rate, %	Chi-square test
Woman’s socioeconomic level						
Familial situation	(*n* = 81,062)			(*n* = 10,769)		
Married or cohabiting	97.9	55.2	188.4^a^	97.7	75.5	58.9^a^
Single	2.1	38.4		2.3	54.2	
Healthcare coverage	(*n* = 80,889)			(*n* = 10,724)		
Insured	98.5	55.1	35.0^a^	98.4	75.3	8.2^a^
Uninsured	1.5	46.6		1.6	65.9	
Education	(*n* = 65,006)			(*n* = 8673)		
Primary school	2.7	21.9	4200.0^a^	1.4	38.8	602.3^a^
Some secondary school	13.8	32.7		10.4	51.9	
Completed secondary school	21.7	45.0		21.1	64.3	
College or university	61.8	63.3		67.1	82.2	
Occupation	(*n* = 57,115)			(*n* = 7906)		
Manual worker	1.3	35.4	1100.0^a^	1.0	60.8	217.6^a^
Office, sales, or service staff	54.7	53.3		58.5	73.7	
Farmer	0.3	40.9		0.2	62.5	
Craft/trades worker or entrepreneur	3.0	56.2		2.7	76.1	
Intermediate (technical)	8.9	59.4		9.2	81.6	
Managerial or higher intellectual	31.8	67.1		28.4	88.5	
Work status	(*n* = 67,502)			(*n* = 8977)		
Working	69.8	60.8	3500.0^a^	75.7	80.7	533.5^a^
Unemployed	6.6	47.4		7.5	62.0	
Not in labor force	23.6	34.0		16.8	53.9	
Partner’s socioeconomic level						
Occupation	(*n* = 63,959)			(*n* = 8454)		
Manual worker	9.1	35.6	2000.0^a^	8.3	57.0	363.7^a^
Office, sales, or service staff	39.7	50.0		44.2	71.1	
Farmer	0.4	43.3		0.3	70.8	
Craft/trades worker or entrepreneur	7.3	51.5		6.5	74.6	
Intermediate (technical)	5.9	58.5		6.7	81.1	
Managerial or higher intellectual	37.6	64.4		34.0	86.3	
Work status	(*n* = 64,121)			(*n* = 8606)		
Working	89.9	56.3	688.3^a^	89.9	77.5	149.5^a^
Unemployed	4.3	37.5		3.9	57.9	
Not in labor force	5.8	40.5		6.2	59.2	

The full sample is composed of all live deliveries performed in the hospitals of the French administrative district of Yvelines in 2008–2014. The low-risk subsample only includes those deliveries of nulliparous women aged 20–34 years, without any diagnosis or co-morbidity, giving birth at full term, by vaginal delivery, without induction, to a singleton infant in cephalic presentation, and with a normal birth weight

^a^ = 1% significance level

### Empirical strategy

To study the effects of both prenatal care and socioeconomic status on cesarean delivery rates, we use the following empirical specification to model the probability of cesarean delivery as:

1 $$ P\ \left({Y}_{ijt}\right)=f\ \left({P}_{ijt},{S}_{ijt}/{X}_{ijt},{V}_{ijt},{e}_{ijt}\ \right) $$

where *i* indexes individuals, *j* hospitals, and *t* years. *Y* is equal to one if the delivery is cesarean and zero otherwise. *P* denotes a vector of observable characteristics of prenatal care use with its clinical, ultrasonographic, and educational components; *S* is a vector of observable socioeconomic conditions: familial situation of the woman, her healthcare coverage, her educational level, her occupation and work status, and her partner’s occupation and work status; *X* is a vector of observable epidemiologic characteristics that affect cesarean delivery use including demographics of the woman: age and parity (i.e., number of previous deliveries), and her medical risk factors: previous cesarean delivery, diabetes, hypertension, eclampsia or preeclampsia, intrauterine growth restriction, placental bleeding, other obstetric pathology (i.e., all diseases or co-morbidities not individually considered such as infection, premature rupture of membranes, obesity, or amniotic fluid abnormality), multiple delivery, term at delivery, fetal presentation, induced labor, and birth weight; *V* denotes a vector of observable characteristics of hospitals, including their type: ownership status, level of equipment assessed based on the level of neonatal care, and teaching status, their organization: day of delivery*,* obstetrician availability, and size expressed as annual volume of deliveries, and their staff: FTEs midwives, obstetricians, and anesthetists per occupied bed; and e is the error term.

To explore whether the effect (if any) of participation in prenatal care on mode of delivery varies by socioeconomic status, we specify the following model of prenatal care utilization:

2 $$ P\ \left({P}_{ijt}\right)=f\ \left({S}_{ijt}/{X}_{ijt},{e}_{ijt}\right) $$

where *i* indexes individuals, *j* hospitals, and *t* years. *P* is a dummy for prenatal care; *X* is a set of individual epidemiologic factors that may impact prenatal care use including demographics of the woman: age and parity, and her medical characteristics: previous cesarean delivery, diabetes, hypertension, eclampsia or preeclampsia, intrauterine growth restriction, placental bleeding, other obstetric pathology, multiple delivery, term at delivery, fetal presentation, onset of labor, mode of delivery, and birth weight; *S* is the set of socioeconomic conditions of the woman and her partner: familial situation, healthcare coverage, educational level, occupation, and work status; and e is the error term.

All models are estimated by multilevel logistic regressions and include heteroscedasticity-robust standard errors that account for within-hospital dependencies across observations. Because our panel consists of women giving birth in different years and different hospitals, we include year of delivery and hospital fixed effects in each model, to capture the heterogeneity in time and among hospitals. Moreover, we also use hospital random effects for model 1, to consider invariant hospital characteristic variables while we assume that the hospital characteristics not explicitly taken into account in the model are not correlated with any explanatory variables.

## Results and discussion

### Descriptive statistics

Table 1 reports the distribution of cesarean deliveries according to prenatal care utilization characteristics. French prenatal care includes at least 7 prenatal visits, which begin during the first trimester. Hence, a woman who has her first prenatal care visit in the second or third trimester starts clinical care late and thus has less prenatal care. Three ultrasounds are recommended: the first trimester ultrasound estimates nuchal translucency thickness to assess the probability of chromosomal abnormalities, while the second or third trimester scan screens for morphologic malformations and anomalies. Prenatal educational services include a prenatal interview offered during the first trimester and prenatal education sessions that usually take place during the third trimester. Cesarean delivery rates do not differ significantly according to the trimester of the first prenatal visit. Among women who have more than the 3 recommended ultrasounds during their pregnancy, 30.4% have cesarean deliveries, significantly higher than the rate around 21% for women who undergo 3 or fewer ultrasounds. Women with no nuchal translucency scan or no morphologic ultrasound have significantly lower cesarean delivery rates, respectively 21.1% and 17%. Women who do not attend an early prenatal interview have a cesarean delivery rate of 23.7%, significantly higher than the 22.3% rate for women who do. Similarly, the cesarean delivery rate for women not attending prenatal education is significantly higher: 24.9% versus 21.9% for those who did. In the subsample of low-risk women, the only difference according to prenatal care use that remains significant in the cesarean delivery rates for this subsample is that based on participation in prenatal education.

Table 1 also shows the variations in the cesarean delivery rates as a function of the socioeconomic characteristics of the women and their partners. Available socioeconomic data apply the official socioeconomic classification of the French national institute of statistics and economic studies (Institut National de la Statistique et des Etudes Economiques (INSEE)) and thus identify the socioeconomic level of each household accurately. The cesarean delivery rates do not differ significantly by family situation or healthcare coverage. However, women with lower educational levels have significantly higher rates of cesarean deliveries: 24.9% for those who only completed primary school, 25.2% for those who did some secondary school, and 25.4% for those who did complete secondary school (i.e., women having reached the final year of secondary school, whether or not they obtained the baccalaureate degree), while the most highly educated women (i.e., post-secondary education) have a cesarean delivery rate of only 22.8%. Similarly, this rate is significantly higher among women working jobs requiring lower skills: 26.7% for manual workers and 24.9% for office, sales, or service staff; compared with higher skills: 23.4% for intermediate (i.e., technical and associate professional) occupations, and 22.7% for managers and higher intellectual workers. The cesarean delivery rate is 25.1% for unemployed women but 24% for working patients. The number of cesarean deliveries also differs according to the partner’s socioeconomic level. Cesarean delivery rates are significantly higher for women whose partners are not working: 24.9% for women with partners that are not in the labor force (i.e., students, apprentices, homemakers, retirees, those on parental leave, and others neither working nor looking for work), and 24.7% when partners are unemployed, whereas 23.6% when partners are working; or have low-skilled jobs: more than 24% when partners have low-skilled jobs, while around 23% when partners have a high-skilled occupation. Moreover, the preliminary statistics for the low-risk subsample are similar, which means epidemiologic factors alone do not explain the socioeconomic factors affecting cesarean delivery use.

As shown above, the only cesarean rates that differ significantly in both the full and low-risk subsamples are those related to participation in prenatal education (Table 1). Moreover, nearly half the women in our entire population do not participate in this care (Table 1). We present these participation rates according to socioeconomic characteristics in Table 2. Single women have a significantly lower participation rate: 38.4% compared with 55.2%. Similarly, uninsured patients participate significantly less often, at rates of 46.6% versus 55.1%, as do women with lower versus higher educational levels. Prenatal education participation rates are 21.9% for women with a primary school education, 32.7% for those with some secondary education, 45% for those who did complete secondary school, and 63.3% for those with some post-secondary education. Similarly, low-skilled women participate at significantly lower rates: 35.4% for manual workers, 40.9% for farmers, and 53.3% for office, sales, or service staff, but 67.1% for women in managerial and higher intellectual/professional occupations. Women out of (versus in) the labor market have significantly lower rates of prenatal education participation: 34% for women not in the labor force, 47.4% for those unemployed, while 60.8% for working women. Results are similar for partners: participation is significantly lower when the partner is not working nor has a low-skilled job. Specifically, the prenatal education participation rate is 35.6% when partners are manual workers, 43.3% when farmers, and 50% when office, sales, or service staff, but 64.4% when they have managerial and higher intellectual occupations; 37.5% and 40.5% when partners are unemployed and out of the labor force, respectively, while 56.3% when they work. The same disparities in prenatal education participation according to socioeconomic variables appear in the low-risk subsample.

### Regression results

We present, first, the effects of regular prenatal care on the cesarean delivery rate. Controlling for epidemiologic and hospital characteristics in columns 1 and 2 of Table 3, we find that prenatal care utilization affects the probability of cesarean deliveries. The period of the first prenatal clinical visit does not affect this probability. Ultrasound care does, however: women undergoing more than 3 ultrasounds have a 31% higher probability of cesarean deliveries. The nuchal translucency ultrasound does not appear to affect cesarean delivery rates. However, women who do not have the morphologic ultrasound have a cesarean delivery probability 25% lower than those who do. Interestingly, women who do not participate in prenatal education are 33% more likely to have cesarean deliveries, although attendance at the early prenatal interview has no clear effect on this probability. When we use the subsample with available socioeconomic variables, which enables us to take these characteristics into account, the results are the same, in both hospital fixed and random effects specifications (columns 5 and 6 of Table 3).

**Table 3:** Effects of prenatal care and socioeconomic status on cesarean delivery use, logit model 1 (odds ratios)

	Full sample						Low-risk subsample			
	(1)	(2)	(3)	(4)	(5)	(6)	(7)	(8)	(9)	(10)
Prenatal care										
First prenatal visit										
Second trimester	1.10 (0.129)	1.09 (0.129)			1.06 (0.174)	1.05 (0.169)	1.75^c^(0.509)	1.67^c^(0.464)	1.40 (0.573)	1.34 (0.524)
Third trimester	1.06 (0.252)	1.05 (0.249)			0.46 (0.259)	0.46 (0.258)	1.92 (1.379)	1.93 (1.386)	2.24 (1.528)	2.27 (1.543)
Obstetric ultrasounds										
< 3	1.02 (0.062)	1.02 (0.061)			1.05 (0.190)	1.05 (0.190)	1.04 (0.329)	1.06 (0.337)	0.87 (0.326)	0.88 (0.334)
≥ 4	1.31^a^(0.057)	1.31^a^(0.058)			1.32^a^(0.090)	1.32^a^(0.091)	1.12 (0.113)	1.11 (0.110)	1.10 (0.121)	1.09 (0.118)
No nuchal translucency ultrasound	0.98 (0.076)	0.99 (0.074)			0.96 (0.130)	0.97 (0.129)	0.95 (0.293)	1.00 (0.289)	1.01 (0.355)	1.06 (0.345)
No morphology ultrasound	0.75^b^(0.108)	0.75^b^(0.108)			0.69^a^(0.076)	0.69^a^(0.076)	0.82 (0.221)	0.78 (0.205)	0.71 (0.235)	0.67 (0.218)
No early prenatal interview	1.06^b^(0.049)	1.06 (0.049)			1.05 (0.048)	1.05 (0.047)	1.00 (0.079)	1.01 (0.077)	0.97 (0.093)	0.98 (0.092)
No prenatal education	1.33^a^(0.022)	1.33^a^(0.022)			1.39^a^(0.038)	1.39^a^(0.038)	1.25^a^(0.068)	1.26^a^(0.062)	1.22^b^(0.098)	1.22^a^(0.092)
Woman’s socioeconomic level										
Single			0.77^a^(0.071)	0.77^a^(0.072)	0.72^c^(0.129)	0.72^c^(0.131)	0.44^a^(0.113)	0.44^a^(0.115)	0.48^a^(0.119)	0.48^a^(0.122)
Uninsured			1.06 (0.078)	1.06 (0.078)	1.10 (0.094)	1.10 (0.094)	0.91 (0.306)	0.92 (0.311)	0.67 (0.247)	0.68 (0.251)
Education										
Primary school			1.23^c^(0.140)	1.22^c^(0.141)	1.19 (0.172)	1.19 (0.173)	1.45 (0.578)	1.45 (0.582)	1.47 (0.603)	1.46 (0.595)
Some secondary school			1.33^a^(0.059)	1.33^a^(0.059)	1.29^a^(0.083)	1.29^a^(0.084)	1.34^b^(0.161)	1.35^b^(0.164)	1.32^b^(0.151)	1.34^b^(0.155)
Completed secondary school			1.29^a^(0.039)	1.29^a^(0.039)	1.24^a^(0.035)	1.24^a^(0.034)	1.27^a^(0.081)	1.28^a^(0.084)	1.29^a^(0.082)	1.30^a^(0.085)
Occupation										
Manual worker			1.26^a^(0.097)	1.26^a^(0.097)	1.33^a^(0.131)	1.33^a^(0.131)	1.55 (0.492)	1.55 (0.495)	1.47 (0.553)	1.50 (0.570)
Office, sales, or service staff			1.13^a^(0.042)	1.13^a^(0.042)	1.12^a^(0.041)	1.12^a^(0.041)	1.14^c^ (0.080)	1.14^c^(0.081)	1.12^c^(0.072)	1.12^c^(0.071)
Farmer			1.24 (0.455)	1.25 (0.457)	1.03 (0.426)	1.04 (0.431)	1.29 (0.877)	1.32 (0.892)	1.80 (1.186)	1.82 (1.194)
Craft/trades worker or entrepreneur			1.10 (0.080)	1.10 (0.080)	1.14^c^(0.090)	1.14^c^(0.090)	1.16 (0.281)	1.15 (0.275)	1.18 (0.354)	1.17 (0.347)
Intermediate (technical)			1.13^a^(0.052)	1.13^a^(0.052)	1.13^c^(0.075)	1.13^c^(0.074)	1.19^a^(0.058)	1.20^a^(0.064)	1.25^a^(0.078)	1.26^a^(0.085)
Work status										
Unemployed			1.14^b^(0.066)	1.14^b^(0.066)	1.14^c^(0.080)	1.14^c^(0.080)	0.81 (0.111)	0.81 (0.114)	0.74^b^(0.099)	0.74^b^(0.101)
Not in labor force			0.94 (0.041)	0.94 (0.041)	0.91 (0.058)	0.91 (0.058)	1.01 (0.111)	1.01 (0.108)	1.01 (0.113)	1.01 (0.116)
Partner’s socioeconomic level										
Occupation										
Manual worker			1.15^a^(0.057)	1.15^a^(0.057)	1.08 (0.067)	1.08 (0.067)				
Office, sales, or service staff			1.13^a^(0.033)	1.13^a^(0.033)	1.11^a^(0.043)	1.11^a^(0.043)				
Farmer			1.06 (0.218)	1.06 (0.217)	1.05 (0.273)	1.05 (0.271)				
Craft/trades worker or entrepreneur			1.10 (0.075)	1.10 (0.074)	1.06 (0.087)	1.06 (0.086)				
Intermediate (technical)			1.07 (0.054)	1.07 (0.055)	1.09 (0.095)	1.09 (0.096)				
Work status										
Unemployed			1.01 (0.080)	1.01 (0.080)	1.11 (0.114)	1.11 (0.115)				
Not in labor force			1.17^a^(0.050)	1.17^a^(0.050)	1.12^b^(0.064)	1.12^b^(0.064)				
Epidemiologic controls	Yes	Yes	Yes	Yes	Yes	Yes	No	No	No	No
Hospital controls	Yes	Yes	Yes	Yes	Yes	Yes	Yes	Yes	Yes	Yes
Year fixed effects	Yes	Yes	Yes	Yes	Yes	Yes	Yes	Yes	Yes	Yes
Hospital effects	Fixed	Random	Fixed	Random	Fixed	Random	Fixed	Random	Fixed	Random
Residence fixed effects	No	No	No	No	No	No	No	No	Yes	Yes
*N* (observations)	68,314	68,314	58,324	58,324	41,141	41,141	9507	9507	8020	8020

Columns 1–6 use the full sample of women while columns 7–10 use the subsample of low-risk women (nulliparous, aged 20–34 years, without any diagnosis or co-morbidity, giving birth at full term, during labor, without induction, to a singleton infant in cephalic presentation, and with a normal birth weight). Epidemiologic control variables include woman’s demographics (age and parity) and medical risk factors (previous cesarean, diabetes, hypertension, eclampsia or preeclampsia, fetal growth restriction, placental bleeding, other obstetric pathology, plurality, term at delivery, fetal presentation, induced labor, and birth weight). Hospital control variables include hospital type (ownership status, equipment level, and teaching status), organization (day of delivery*,* obstetrician availability, and size), and staff (midwives, obstetricians, and anesthetists in FTEs per bed). Hospital invariant control variables (ownership status, equipment level, and teaching status) are only included in regressions with hospital random effects. Robust standard errors in parentheses

^a^ = 1% significance level, ^b^ = 5%, ^c^ = 10%

Table 3 also shows in columns 3 and 4 the effects of the socioeconomic status of each partner on the use of cesarean deliveries while controlling for epidemiologic and hospital characteristics. Familial situation has a significant effect: single women are 23% more likely to have a cesarean delivery compared to those who do not. Insurance coverage, however, does not affect this probability. More interestingly, compared with the most highly educated women, patients with primary, some secondary, or completed secondary education are respectively 22–23%, 33% and 29% more likely to have a cesarean delivery. Similarly, women working at low-skilled jobs have a higher probability of cesarean deliveries: compared with the most highly qualified women, this probability is 26% higher for manual workers, and 13% higher for office, sales, or service staff as well as for workers with intermediate occupations. Moreover, unemployed women have a probability of a cesarean delivery 14% higher than that of working women. Likewise, the partner’s socioeconomic level affects the woman’s probability of a cesarean delivery, which is 15% higher for women whose partners are manual workers, and 13% higher when they are service workers. This probability is 17% higher when the partner is not in the labor force, versus is working. Adjustment for hospitals’ fixed or random effects does not change these results, which are similar even in the subsample with available prenatal care variables (columns 5 and 6 of Table 3). According to our study, low socioeconomic status significantly and substantially increases the probability of cesarean deliveries. Our results are therefore consistent with the previous literature from high-income countries, as detailed above in the Introduction.

Table 6 in Appendix presents the effects of the epidemiologic and hospital control variables on cesarean delivery use. Many epidemiologic control risk factors are significantly associated with a higher probability of cesarean deliveries: age, nulliparity, previous cesarean, diabetes, hypertension, eclampsia or preeclampsia, fetal growth restriction, placental bleeding, other obstetric pathology, preterm and post-term delivery, abnormal presentation, induced labor, low and high birth weight; as are some hospital control characteristics: private ownership status, high equipment level, working-day delivery, low hospital size, and low FTE obstetricians per bed. As expected, the effects of these variables are similar to those found in previous studies.

Our results clearly demonstrate that, all else being equal, both prenatal care and socioeconomic position influence the cesarean decision. Because the data have been anonymized, women are not identifiable within the study period. We cannot thus use the fixed effects for women that would allow us to control for the unobservable heterogeneity between them, which may correlate with cesarean delivery use and either prenatal care utilization or socioeconomic status. However, we do control for many observed characteristics of women and their partners. Furthermore, we also performed several additional sensitivity analyses to consider potential relevant confounding factors. First, one natural and straightforward explanation of our results might be the epidemiologic factors: the low-income women and those with high utilization rates of ultrasound care or low rates of prenatal educational care may also be the at-risk population. For example, women with various epidemiologic risk factors require more obstetric ultrasounds than traditionally recommended. Furthermore, the low-income population is also the population most at risk in terms of co-morbidity and diagnosis. As a robustness check, we examine the low-risk subsample to further control for medical severity (see details on the low-risk subsample for cesarean delivery use above in the Methods). These results are presented in columns 7 and 8 of Table 3. In this subsample with fewer observations, we include woman’s socioeconomic characteristics only, to avoid the consequences of the very likely collinearity between the socioeconomic variables of the woman and her partner on the efficiency of the estimates. When we focus on low-risk women, the effects of the obstetric ultrasound care are no longer significant, but the effects of prenatal education remain significantly associated with a higher probability of a cesarean delivery for non-participants. In addition, the woman’s socioeconomic position has the same effects on the cesarean delivery probability in this subsample as in the overall population: the lower the socioeconomic indicator, the higher the cesarean delivery rate. Using the partner’s socioeconomic variables produces similar findings (Table 7 in Appendix).

Woman’s preferences are another important factor to consider: those who choose to undergo substantial ultrasonographic examinations as well as those who prefer not to attend prenatal education may also prefer a cesarean delivery. To control for the woman’s preference, we can use unplanned deliveries, assuming that if the woman has a preference for a cesarean delivery, the obstetrician would plan this. The data available here do not provide any information about women’s preferences. This assumption can be debated, but appears reasonable to us. The existing literature reports that women’s preferences affect the rates of elective cesarean deliveries [19, 46–48]. Reasons identified as influencing a woman’s decision to request an elective cesarean delivery include cultural factors, fear of pain during labor and delivery, previous experience, and interactions with health care professionals [47]. The woman’s choice can be a primary indication for planned elective cesarean deliveries, but is not an indication for those that are not planned [48].

Unplanned deliveries also include only deliveries for which the patient did not know her mode of delivery in advance. Because most women still perceive prenatal education simply as preparation for vaginal delivery, women with a planned cesarean delivery may choose not to attend. Focusing on unplanned deliveries may thus control for these two factors. Columns 7 and 8 of Table 3 report the effects of prenatal care utilization on cesarean delivery use in the low-risk subsample that includes only unplanned deliveries. Even when the mode of delivery is not planned, prenatal education is still a highly significant variable. Therefore, neither women’s preference nor their advance knowledge of their mode of delivery explains the effects of prenatal education on cesarean deliveries.

Furthermore, income level is a factor that explains woman’s access to prenatal care [25, 49], and women with low socioeconomic positions may face constraints in their access to health care that may explain their high use of cesarean deliveries. Since no healthcare access variable is available in our data, we use the woman’s town of residence, available for some observations. We do not include town of residence fixed effects in the first regressions because this variable is not available for all observations. Columns 9 and 10 of Table 3 show the effects of socioeconomic status on the probability of a cesarean delivery for low-risk women, with dummy variables included for town of residence. Cesarean delivery is still most prevalent for women with low, compared with high, socioeconomic status. We therefore cannot interpret lack of access to obstetric care as the explanation for the effects of socioeconomic status on cesarean delivery use. Moreover, including fixed effects for town of residence also allows us to control for area-based socioeconomic differences. Individual and area-based socioeconomic conditions are different aspects of socioeconomic position, and both dimensions may affect the probability of a cesarean delivery [50]. Indeed, individual socioeconomic characteristics may capture some effects of area-based socioeconomic conditions, as women with low socioeconomic positions mostly live in less affluent areas. When we include dummy variables for the woman’s town of residence, the effects of socioeconomic status on the probability of cesarean deliveries persist. Individual socioeconomic status is thus an independent socioeconomic factor affecting cesarean delivery rates. The results are again the same even when we use the partner’s socioeconomic characteristics (Table 7 in Appendix).

Because prenatal education participation significantly affects cesarean deliveries, we sought to assess in column 1 of Table 4 whether or not socioeconomic conditions affect its utilization during pregnancy, while taking epidemiologic characteristics into account. Familial situation and healthcare coverage do not significantly affect the probability of this participation, but educational level does. Specifically, compared to the women with the most education, the probability of attendance at prenatal education for those with primary schooling only, some secondary school, and who completed secondary school is respectively 47%, 42%, and 27% lower. Likewise, the women with the fewest skills are least likely to participate in prenatal education. Compared with the managers (i.e., the most highly skilled women), women who are crafts/trades workers or entrepreneurs are 13% less likely to participate, and those working as office, sales, or service staff 11% less likely. Further, compared with working women, those who are not in the labor force as well as unemployed ones are respectively 29% and 14% less likely to participate. The same is true for women whose partners have low-skilled jobs or do not work: the probability of participation is 26% lower when the partner is a manual worker or a farmer, and 15% lower if he is an office, sales, or service worker, as well as 26% lower if unemployed, and 22% lower if he is not in the labor force.

**Table 4:** Effects of socioeconomic status on prenatal education utilization, logit model 2 (odds ratios)

	Full sample	Low-risk subsample	
	(1)	(2)	(3)
Woman’s socioeconomic level			
Single	1.07 (0.134)	0.52^a^(0.087)	0.50^a^(0.088)
Uninsured	1.06 (0.111)	1.37 (0.475)	1.34 (0.578)
Education			
Primary school	0.53^a^(0.106)	0.61 (0.189)	0.72 (0.221)
Some secondary school	0.58^a^(0.054)	0.43^a^(0.088)	0.45^a^(0.117)
Completed secondary school	0.73^a^(0.038)	0.62^a^(0.086)	0.60^a^(0.098)
Occupation			
Manual worker	0.90 (0.128)	0.63 (0.279)	0.45 (0.231)
Office, sales, or service staff	0.89^a^(0.023)	0.65^a^(0.041)	0.63^a^(0.049)
Farmer	0.75 (0.170)	0.49 (0.358)	0.51 (0.382)
Craft/trades worker or entrepreneur	0.87^a^(0.046)	0.58^a^(0.062)	0.60^a^(0.081)
Intermediate (technical)	0.94 (0.061)	0.84 (0.152)	0.77^c^(0.118)
Work status			
Unemployed	0.86^a^(0.032)	0.77^c^(0.107)	0.80^c^(0.095)
Not in labor force	0.71^a^(0.034)	0.57^a^(0.043)	0.62^a^(0.080)
Partner’s socioeconomic level			
Occupation			
Manual worker	0.74^a^(0.050)		
Office, sales, or service staff	0.85^a^(0.027)		
Farmer	0.74^b^(0.109)		
Craft/trades worker or entrepreneur	0.91^c^(0.045)		
Intermediate (technical)	0.92^b^(0.039)		
Work status			
Unemployed	0.74^a^(0.051)		
Not in labor force	0.78^a^(0.052)		
Epidemiologic controls	Yes	No	No
Year fixed effects	Yes	Yes	Yes
Hospital effects	Fixed	Fixed	Fixed
Residence fixed effects	No	No	Yes
*N* (observations)	48,042	7064	6033

Column 1 uses the full sample of women while columns 2 and 3 use the subsample of low-risk women (nulliparous, aged 20–34 years, without any diagnosis or co-morbidity, giving birth at full term, by vaginal delivery, without induction, to a singleton infant in cephalic presentation, and with a normal birth weight). Epidemiologic control variables include woman’s demographics (age and parity) and medical factors (previous cesarean, diabetes, hypertension, eclampsia or preeclampsia, fetal growth restriction, placental bleeding, other obstetric pathology, plurality, term at delivery, fetal presentation, onset of labor, mode of delivery, and birth weight). Robust standard errors in parentheses

^a^ = 1% significance level, ^b^ = 5%, ^c^ = 10%

Table 8 in Appendix presents the effects of the epidemiologic control variables on prenatal education participation. Several epidemiologic control factors are significantly associated with a lower probability of prenatal education attendance: younger and older age, multiparity, diabetes, fetal growth restriction, other obstetric pathology, preterm delivery, non-spontaneous onset of labor, cesarean delivery, and low birth weight.

Again, to better control for differences in epidemiologic characteristics that may explain these findings, we perform the same estimates in the low-risk subsample (see details on the low-risk subsample for prenatal care participation above in the Methods). The results are presented in column 2 of Table 4. When we focus on low-risk patients, women in low socioeconomic positions remain less likely to participate in prenatal education. Moreover, we also include the woman’s town of residence as a fixed effect in column 3 of Table 4, which made it possible to take geographic differences across women into account, including those related to health care access and to area-based socioeconomic status. Indeed, individual socioeconomic variables can capture disparities in healthcare access as well as the variables based on area-based socioeconomic conditions. Our finding show that, regardless of differences in prenatal care access and area-based socioeconomic situation, women with low, compared with high, socioeconomic status are less likely to use prenatal education. Results using partner’s socioeconomic variables are very similar (Table 9 in Appendix).

Because our results show that socioeconomic status significantly affects prenatal education participation (Table 4), we went on to examine the effects of interaction terms for both of these variables on cesarean delivery use. We thus study whether or not the effects of prenatal education vary by socioeconomic status, which we are unable to study from the results of Table 3. For each occupation, we seek to compare the impact of socioeconomic status on those with and without prenatal education. Therefore, we add dummy variables for prenatal education crossed with the dummy variables for the woman’s occupation, as well as dummy variables for no prenatal education crossed with the dummy variables for the woman’s occupation. This exclude from the regression the global effects of prenatal education and of occupation type; when we consider all types of occupation and the constant, however, it introduce a strict collinearity problem. We therefore include a constraint in the model: the sum of all the crossed occupation and prenatal education variables equals 0. The results are presented as odds ratios. Table 10 in Appendix provides the results as coefficients. This model also enables us to answer the question: Do the effects of prenatal education vary for different occupation types? We do so by comparing the coefficient values between different occupation types crossed with prenatal education. Table 5 reports the results for model 1 with the variables described here. In low and intermediate occupational category (manual workers, office, sales, or service staff, and intermediate (technical) occupations), the women who do not participate in prenatal education are more likely to have cesarean deliveries than those who do participate. Moreover, in response to the question of whether the effects of prenatal education vary for different occupation types, we see that for managerial or higher intellectual occupations as well as intermediate (technical) occupations, prenatal education has a negative cumulative effect with socioeconomic status on the probability of cesarean deliveries (compared to the average effect of the sample). Interaction terms for prenatal education and other available socioeconomic variables yield the same results (Table 11 in the Appendix presents the results as odds ratios. However, the results as coefficients are available upon request).

**Table 5:** Effects of prenatal care and socioeconomic status on cesarean delivery use, interaction terms for prenatal care and socioeconomic status, logit model 1 (odds ratios)

	(1)	(2)
Crossed dummy variables for woman’s occupation and prenatal education participation		
Manual worker × No prenatal education	1.28^b^(0.131)	1.28^b^(0.130)
Manual worker × Prenatal education	1.16 (0.196)	1.16 (0.198)
Office, sales, or service staff × No prenatal education	1.16^c^(0.097)	1.16^c^(0.097)
Office, sales, or service staff × Prenatal education	0.86^c^(0.069)	0.85^c^(0.069)
Farmer × No prenatal education	1.10 (0.621)	1.11 (0.629)
Farmer × Prenatal education	0.76 (0.227)	0.76 (0.228)
Craft/trades worker or entrepreneur × No prenatal education	1.11 (0.187)	1.11 (0.187)
Craft/trades worker or entrepreneur × Prenatal education	0.91 (0.131)	0.90 (0.131)
Intermediate (technical) occupation × No prenatal education	1.20^b^(0.102)	1.20^b^(0.101)
Intermediate (technical) occupation × Prenatal education	0.84^a^(0.050)	0.84^a^(0.050)
Managerial or higher intellectual occupation × No prenatal education	1.10 (0.067)	1.10 (0.066)
Managerial or higher intellectual occupation × Prenatal education	0.74^a^(0.060)	0.74^a^(0.059)
Epidemiologic and hospital controls	Yes	Yes
Other prenatal care and socioeconomic variables	Yes	Yes
Year fixed effects	Yes	Yes
Hospital effects	Fixed	Random
*N* (observations)	41,141	41,141

All regressions use the full sample of women and include a constraint: the sum of all the crossed variables equals 0. Epidemiologic control variables include woman’s demographics (age and parity) and medical risk factors (previous cesarean, diabetes, hypertension, eclampsia or preeclampsia, fetal growth restriction, placental bleeding, other obstetric pathology, plurality, term at delivery, fetal presentation, induced labor, and birth weight). Hospital control variables include hospital type (ownership status, equipment level, and teaching status), organization (day of delivery*,* obstetrician availability, and size), and staff (midwives, obstetricians, and anesthetists in FTEs per bed). Hospital invariant control variables (ownership status, equipment level, and teaching status) are only included in the regression with hospital random effects. Other prenatal care variables are trimester of the first antenatal visit, number of obstetric ultrasounds, nuchal translucency ultrasound, morphology ultrasound, and early prenatal interview. Other socioeconomic variables include woman’s familial situation, healthcare coverage, education and work status, and her partner’s occupation and work status. Robust standard errors in parentheses

^a^ = 1% significance level, ^b^ = 5%, ^c^ = 10%

### Discussion

We investigate a rarely studied question in this paper: how patient care throughout pregnancy affects mode of delivery. We use a rich and large database of delivery information for the years 2008–2014, which allows us to take many patient- and hospital-level characteristics that may affect obstetric practices into account. We estimate multilevel logit models, first to study the effects of prenatal care on cesarean delivery and then to assess whether socioeconomic status influences prenatal care and specifically participation in prenatal education, which appears to affect mode of delivery significantly.

Our primary results show that prenatal education affects cesarean delivery rates. The probability of a cesarean delivery increases by 20 to 40% for women who do not participate in prenatal education. Two mechanisms may explain this finding. On the one hand, prenatal education may improve women’s knowledge about mode of delivery. Several studies report that women are not aware of the risks and benefits of birth procedures, and that this lack of knowledge results in some of them choosing cesarean rather than vaginal delivery [51, 52]. Indeed, a substantial proportion of women continue to consider the cesarean as the safest method of delivery, especially for the child, even though epidemiologic studies demonstrate conclusively that cesarean, compared to vaginal, deliveries increase maternal morbidity in this and future pregnancies and do not improve infant health. On the other hand, prenatal education may have positive psychological effects on pregnant women, who are often anxious about giving birth. Indeed, fear of childbirth is strongly associated with performance of cesarean deliveries [53].

Since a significant proportion of obstetricians are willing to proceed with a cesarean delivery if requested [54, 55], the woman’s choice is an important determinant of cesarean deliveries to consider: the number of patient-request cesarean deliveries is currently estimated at 4–18% of the total [56]. Women attending prenatal education may be more aware of the risks of cesarean deliveries and less affected by fear of giving birth, and may therefore ask less often for a cesarean delivery. Moreover, a well-informed patient is likely to be better able to respond to the information she receives from the obstetrician and to participate in the decision process about method of delivery than someone less informed. This may affect physician and hospital incentives to perform more cesarean deliveries.

Moreover, we find that socioeconomic status influences uptake of prenatal education. Using several individual socioeconomic indicators, our results confirm that low socioeconomic women are more likely to have cesarean deliveries and further show that women in this subgroup have a lower probability of participating in prenatal education. For example, compared to the women with the most education, women who have no post-secondary schooling have at least a 20% lower probability of attendance at prenatal education. The problem of socioeconomic disparities in method of delivery is the focus of much attention, especially in view of the difficulty in modifying socioeconomic differences by public policy interventions. Since utilization of prenatal education may be a factor substantially more susceptible to change, our finding is of interest.

In order to address the possible individual level self-selection into prenatal education participation, our empirical model includes a large set of available covariates. We also perform several robustness analyses that allow many potential confounding factors to be taken into account. However, we cannot fully control for all differences between women who choose to attend prenatal education and women who do not, including in their risk for cesarean delivery. Hence, our results do not allow any causal inferences. Future research exploring the effect of implementation or promotion of such care programs on mode of delivery is thus highly recommended. If our observed associations are confirmed to be causal, a straightforward implication would be that public policies promoting participation rates for prenatal education and targeting this promotion primarily at low-income women could lead to real reductions in cesarean delivery rates.

## Conclusion

Overall, our analysis reinforces recent economic studies reporting that support of patient decision-making may impact the use of medical services substantially. Previous efforts to limit inappropriate medical care has mainly targeted providers, for instance, by reducing benefits or restricting eligibility, but has encountered serious political difficulties. Patient education may have an important impact on limiting medical overuse.

## Appendix

**Table 6:** Effects of epidemiologic and hospital factors on cesarean delivery use, logit model 1 (odds ratios)

	(1)	(2)	(3)	(4)	(5)	(6)
Patient demographics						
Age (years)	1.05^a^ (0.002)	1.05^a^ (0.002)	1.06^a^ (0.003)	1.06^a^ (0.003)	1.06^a^ (0.002)	1.06^a^ (0.002)
Nulliparous	3.57^a^ (0.283)	3.57^a^ (0.283)	3.34^a^ (0.250)	3.33^a^ (0.250)	3.82^a^ (0.277)	3.81^a^ (0.277)
Medical risk factors						
Previous cesarean	20.60^a^ (1.934)	20.59^a^ (1.928)	21.75^a^ (2.560)	21.73^a^ (2.548)	22.06^a^ (2.448)	22.03^a^ (2.436)
Diabetes	1.37^a^ (0.080)	1.37^a^ (0.081)	1.26^b^ (0.130)	1.26^b^ (0.130)	1.26^a^ (0.107)	1.26^a^ (0.107)
Hypertension	1.91^a^ (0.116)	1.91^a^ (0.115)	1.67^a^ (0.151)	1.67^a^ (0.151)	1.75^a^ (0.176)	1.75^a^ (0.175)
Eclampsia or preeclampsia	3.59^a^ (0.279)	3.59^a^ (0.280)	3.63^a^ (0.631)	3.63^a^ (0.631)	4.22^a^ (0.568)	4.23^a^ (0.570)
Fetal growth restriction	1.80^a^ (0.254)	1.80^a^ (0.254)	2.18^a^ (0.529)	2.18^a^ (0.529)	2.10^a^ (0.483)	2.11^a^ (0.484)
Placental bleeding	10.49^a^ (3.541)	10.52^a^ (3.545)	11.67^a^ (3.684)	11.73^a^ (3.682)	13.42^a^ (4.661)	13.46^a^ (4.657)
Other obstetric pathology	0.98 (0.028)	0.98 (0.028)	1.08^a^ (0.030)	1.08^a^ (0.029)	1.03 (0.035)	1.03 (0.035)
Multiple delivery	1.11 (0.187)	1.11 (0.187)	1.31 (0.242)	1.30 (0.242)	1.24 (0.230)	1.24 (0.230)
Term at delivery						
Preterm (< 37 weeks of gestation)	1.39^a^ (0.143)	1.39^a^ (0.143)	1.37^a^ (0.129)	1.38^a^ (0.129)	1.27^b^ (0.155)	1.27^b^ (0.154)
Post-term (> 41 weeks of gestation)	2.38^a^ (0.547)	2.40^a^ (0.549)	3.18^a^ (0.629)	3.19^a^ (0.625)	2.89^a^ (0.681)	2.90^a^ (0.679)
Abnormal presentation (Breech or transverse)	33.19^a^ (4.784)	33.17^a^ (4.777)	33.99^a^ (4.554)	33.97^a^ (4.546)	36.40^a^ (6.517)	36.37^a^ (6.498)
Induced labor	1.16^b^ (0.088)	1.16^b^ (0.088)	1.15^c^ (0.093)	1.15^c^ (0.093)	1.14^c^ (0.090)	1.14^c^ (0.090)
Birth weight						
Low birth weight (< 2500 g)	1.69^a^ (0.095)	1.69^a^ (0.095)	1.65^a^ (0.094)	1.65^a^ (0.094)	1.49^a^ (0.115)	1.49^a^ (0.114)
High birth weight (> 4000 g)	1.95^a^ (0.147)	1.95^a^ (0.147)	2.08^a^ (0.190)	2.08^a^ (0.190)	2.07^a^ (0.168)	2.07^a^ (0.169)
Hospital type						
Private		1.79^b^ (0.419)		1.79^a^ (0.326)		2.21^a^ (0.402)
Level of equipment						
Neonatology unit		1.26^b^ (0.137)		1.22^b^ (0.110)		1.31^a^ (0.124)
Neonatal intensive care unit		1.52^b^ (0.318)		1.46^b^ (0.246)		1.65^a^ (0.245)
Teaching		1.04 (0.162)		1.14 (0.140)		1.17 (0.121)
Hospital organization						
Non-working day delivery (weekend or holiday)	0.61^a^ (0.029)	0.61^a^ (0.029)	0.59^a^ (0.029)	0.59^a^ (0.029)	0.60^a^ (0.032)	0.60^a^ (0.032)
On-call obstetrician outside the unit	1.13 (0.105)	0.95 (0.099)	1.26^b^ (0.126)	1.04 (0.106)	1.17 (0.123)	0.88 (0.095)
Size						
< 1000 deliveries per year	0.99 (0.043)	1.04 (0.050)	0.83^a^ (0.033)	1.02 (0.117)	1.17^a^ (0.044)	1.25^c^ (0.145)
≥ 2000 deliveries per year	0.93^c^ (0.038)	0.92^b^ (0.035)	0.94 (0.045)	0.93^c^ (0.039)	0.91^b^ (0.040)	0.90^a^ (0.034)
Hospital staff						
Midwives (FTEs per bed)	0.87 (0.104)	0.87 (0.097)	0.89 (0.132)	0.90 (0.126)	0.88 (0.124)	0.91 (0.112)
Obstetricians (FTEs per bed)	0.70 (0.185)	0.68^c^ (0.144)	0.57^c^ (0.183)	0.57^b^ (0.154)	0.69 (0.229)	0.70 (0.172)
Anesthetists (FTEs per bed)	1.25 (0.480)	1.21 (0.386)	1.06 (0.354)	1.06 (0.257)	1.10 (0.450)	1.05 (0.296)
Prenatal care variables	Yes	Yes	No	No	Yes	Yes
Socioeconomic variables	No	No	Yes	Yes	Yes	Yes
Year fixed effects	Yes	Yes	Yes	Yes	Yes	Yes
Hospital effects	Fixed	Random	Fixed	Random	Fixed	Random
*N* (observations)	68,314	68,314	58,324	58,324	41,141	41,141

All regressions use the full sample of women. Prenatal care variables are trimester of the first antenatal visit, number of obstetric ultrasounds, nuchal translucency ultrasound, morphology ultrasound, early prenatal interview, and prenatal education. Socioeconomic variables include woman’s socioeconomic level (familial situation, healthcare coverage, education, occupation, and work status), and her partner’s one (occupation and work status). Robust standard errors in parentheses

^a^ = 1% significance level, ^b^ = 5%, ^c^ = 10%

**Table 7:** Effects of prenatal care and socioeconomic status on cesarean delivery use, low-risk subsample with partner’s socioeconomic variables included, logit model 1 (odds ratios)

	(1)	(2)	(3)	(4)
Prenatal care				
First prenatal visit				
Second trimester	1.09 (0.299)	1.03 (0.271)	0.69 (0.356)	0.67 (0.284)
Third trimester	3.69^b^ (1.907)	3.79^a^ (1.921)	3.46^b^ (1.716)	3.58^a^ (1.704)
Obstetric ultrasounds				
< 3	0.83 (0.301)	0.84 (0.307)	0.71 (0.310)	0.72 (0.245)
≥ 4	1.14^c^ (0.088)	1.12 (0.087)	1.12 (0.090)	1.10 (0.087)
No nuchal translucency ultrasound	0.89 (0.210)	0.93 (0.202)	0.97 (0.281)	1.01 (0.232)
No morphology ultrasound	0.84 (0.314)	0.80 (0.295)	0.79 (0.327)	0.75 (0.218)
No early prenatal interview	1.01 (0.083)	1.02 (0.079)	0.99 (0.080)	1.01 (0.090)
No prenatal education	1.35^a^ (0.082)	1.37^a^ (0.084)	1.33^a^ (0.112)	1.33^a^ (0.088)
Partner’s socioeconomic level				
Occupation				
Manual worker	0.99 (0.182)	1.00 (0.187)	0.88 (0.127)	0.89 (0.195)
Office, sales, or service staff	1.09 (0.089)	1.10 (0.090)	1.08 (0.088)	1.08 (0.077)
Farmer	2.14^b^ (0.798)	2.14^b^ (0.794)	2.57^b^ (1.179)	2.56^b^ (1.005)
Craft/trades worker or entrepreneur	1.05 (0.121)	1.05 (0.121)	1.01 (0.152)	1.02 (0.142)
Intermediate (technical)	0.78 (0.143)	0.79 (0.142)	0.84 (0.132)	0.85 (0.162)
Work status				
Unemployed	1.44^a^ (0.118)	1.43^a^ (0.115)	1.60^b^ (0.293)	1.60^a^ (0.169)
Not in labor force	0.90 (0.081)	0.89 (0.082)	0.88 (0.153)	0.86 (0.096)
Hospital controls	Yes	Yes	Yes	Yes
Year fixed effects	Yes	Yes	Yes	Yes
Hospital effects	Fixed	Random	Fixed	Random
Residence fixed effects	No	No	Yes	Yes
*N* (observations)	10,732	10,732	9197	9197

All regressions use the subsample of low-risk women (nulliparous, aged 20–34 years, without any diagnosis or co-morbidity, giving birth at full term, during labor, without induction, to a singleton infant in cephalic presentation, and with a normal birth weight). Hospital control variables include hospital type (ownership status, equipment level, and teaching status), organization (day of delivery*,* obstetrician availability, and size), and staff (midwives, obstetricians, and anesthetists in FTEs per bed). Hospital invariant control variables (ownership status, equipment level, and teaching status) are only included in regressions with hospital random effects. Robust standard errors in parentheses

^a^ = 1% significance level, ^b^ = 5%, ^c^ = 10%

**Table 8:** Effects of epidemiologic characteristics on prenatal care utilization, logit model 2 (odds ratios)

	(1)
Patient demographics	
Age	
< 20 years	0.33^a^ (0.041)
**≥** 35 years	0.94^a^ (0.021)
Parity	
= 1	0.26^a^ (0.035)
≥ 2	0.14^a^ (0.024)
Medical factors	
Previous cesarean	0.96 (0.047)
Diabetes	0.81^a^ (0.046)
Hypertension	1.05 (0.126)
Eclampsia or preeclampsia	0.92 (0.114)
Fetal growth restriction	0.83^c^ (0.092)
Placental bleeding	1.05 (0.154)
Other obstetric pathology	0.76^a^ (0.035)
Multiple delivery	0.97 (0.119)
Term at delivery	
Preterm (< 37 weeks of gestation)	0.64^a^ (0.030)
Post-term (> 41 weeks of gestation)	1.08 (0.337)
Abnormal presentation (Breech or transverse)	0.99 (0.128)
Onset of labor	
Induced	0.91^b^ (0.036)
Cesarean before labor	0.63^a^ (0.016)
Mode of delivery	
Cesarean	0.87^a^ (0.030)
Birth weight	
Low birth weight (< 2500 g)	0.80^a^ (0.064)
High birth weight (> 4000 g)	1.01 (0.042)
Socioeconomic variables	Yes
Year fixed effects	Yes
Hospital effects	Fixed
*N* (observations)	48,042

The regression uses the full sample of women. Socioeconomic variables include woman’s socioeconomic level (familial situation, healthcare coverage, education, occupation, and work status), and her partner’s one (occupation and work status). Robust standard errors in parentheses

^a^ = 1% significance level, ^b^ = 5%, ^c^ = 10%

**Table 9:** Effects of socioeconomic status on prenatal education utilization, low-risk subsample with partner’s socioeconomic variables included, logit model 2 (odds ratios)

	(1)	(2)
Partner’s socioeconomic level		
Occupation		
Manual worker	0.37^a^ (0.086)	0.36^a^ (0.076)
Office, sales, or service staff	0.53^a^ (0.062)	0.53^a^ (0.060)
Farmer	0.46^b^ (0.156)	0.62 (0.193)
Craft/trades worker or entrepreneur	0.61^b^ (0.124)	0.62^b^ (0.118)
Intermediate (technical)	0.90 (0.147)	0.90 (0.178)
Work status		
Unemployed	0.61^a^ (0.084)	0.63^a^ (0.080)
Not in labor force	0.57^a^ (0.055)	0.60^a^ (0.081)
Year fixed effects	Yes	Yes
Hospital effects	Fixed	Fixed
Residence fixed effects	No	Yes
*N* (observations)	8083	6977

All regressions use the subsample of low-risk women (nulliparous, aged 20–34 years, without any diagnosis or co-morbidity, giving birth at full term, by vaginal delivery, without induction, to a singleton infant in cephalic presentation, and with a normal birth weight). Robust standard errors in parentheses

^a^ = 1% significance level, ^b^ = 5%

**Table 10:** Effects of prenatal care and socioeconomic status on cesarean delivery use, interaction terms for prenatal care and socioeconomic status, logit model 1 (coefficients)

	(1)	(2)
Crossed dummy variables for woman’s occupation and prenatal education participation		
Manual worker × No prenatal education	0.25^b^ (0.102)	0.24^b^ (0.102)
Manual worker × Prenatal education	0.14 (0.170)	0.15 (0.171)
Office, sales, or service staff × No prenatal education	0.15^c^ (0.084)	0.15^c^ (0.083)
Office, sales, or service staff × Prenatal education	−0.16^c^ (0.081)	−0.16^c^ (0.081)
Farmer × No prenatal education	0.10 (0.565)	0.11 (0.566)
Farmer × Prenatal education	−0.28 (0.301)	− 0.28 (0.300)
Craft/trades worker or entrepreneur × No prenatal education	0.10 (0.169)	0.10 (0.169)
Craft/trades worker or entrepreneur × Prenatal education	−0.10 (0.144)	−0.10 (0.145)
Intermediate (technical) occupation × No prenatal education	0.18^b^ (0.085)	0.18^b^ (0.084)
Intermediate (technical) occupation × Prenatal education	−0.17^a^ (0.060)	−0.18^a^ (0.060)
Managerial or higher intellectual occupation × No prenatal education	0.09 (0.061)	0.09 (0.060)
Managerial or higher intellectual occupation × Prenatal education	−0.30^a^ (0.081)	−0.31^a^ (0.081)
Epidemiologic and hospital controls	Yes	Yes
Other prenatal care and socioeconomic variables	Yes	Yes
Year fixed effects	Yes	Yes
Hospital effects	Fixed	Random
*N* (observations)	41,141	41,141

All regressions use the full sample of women and include a constraint: the sum of all the crossed variables equals 0. Epidemiologic control variables include woman’s demographics (age and parity) and medical risk factors (previous cesarean, diabetes, hypertension, eclampsia or preeclampsia, fetal growth restriction, placental bleeding, other obstetric pathology, plurality, term at delivery, fetal presentation, induced labor, and birth weight). Hospital control variables include hospital type (ownership status, equipment level, and teaching status), organization (day of delivery*,* obstetrician availability, and size), and staff (midwives, obstetricians, and anesthetists in FTEs per bed). Hospital invariant control variables (ownership status, equipment level, and teaching status) are only included in the regression with hospital random effects. Other prenatal care variables are trimester of the first antenatal visit, number of obstetric ultrasounds, nuchal translucency ultrasound, morphology ultrasound, and early prenatal interview. Other socioeconomic variables include woman’s familial situation, healthcare coverage, education and work status, and her partner’s occupation and work status. Robust standard errors in parentheses

^a^ = 1% significance level, ^b^ = 5%, ^c^ = 10%

**Table 11:** Effects of prenatal care and socioeconomic status on cesarean delivery use, further interaction terms for prenatal care and socioeconomic status, logit model 1 (odds ratios)

	(1)	(2)	(3)	(4)	(5)	(6)	(7)	(8)
Crossed dummy variables for woman’s education and prenatal education participation								
Primary school × No prenatal education	1.01 (0.137)	1.01 (0.137)						
Primary school × Prenatal education	1.12 (0.278)	1.12 (0.281)						
Some secondary school × No prenatal education	1.16^b^ (0.071)	1.16^b^ (0.071)						
Some secondary school × Prenatal education	1.03 (0.047)	1.03 (0.047)						
Completed secondary school × No prenatal education	1.14^b^ (0.067)	1.14^b^ (0.067)						
Completed secondary school × Prenatal education	0.92 (0.051)	0.92 (0.053)						
College or university × No prenatal education	1.03 (0.053)	1.03 (0.052)						
College or university × Prenatal education	0.68^a^ (0.023)	0.68^a^ (0.023)						
Crossed dummy variables for woman’s work status and prenatal education participation								
Unemployed × No prenatal education			1.28^a^ (0.083)	1.28^a^ (0.084)				
Unemployed × Prenatal education			0.98 (0.079)	0.98 (0.078)				
Not in labor force × No prenatal education			0.95 (0.057)	0.95 (0.056)				
Not in labor force × Prenatal education			0.86 (0.094)	0.87 (0.094)				
Working × No prenatal education			1.19^a^ (0.036)	1.19^a^ (0.035)				
Working × Prenatal education			0.82^a^ (0.019)	0.82^a^ (0.019)				
Crossed dummy variables for partner’s occupation and prenatal education participation								
Manual worker × No prenatal education					1.11 (0.073)	1.11^c^ (0.072)		
Manual worker × Prenatal education					0.94 (0.062)	0.94 (0.063)		
Office, sales, or service staff × No prenatal education					1.22^a^ (0.092)	1.22^a^ (0.091)		
Office, sales, or service staff × Prenatal education					0.88^a^ (0.039)	0.88^a^ (0.039)		
Farmer × No prenatal education					1.07 (0.185)	1.06 (0.186)		
Farmer × Prenatal education					0.91 (0.365)	0.90 (0.362)		
Craft/trades worker or entrepreneur × No prenatal education					1.09 (0.098)	1.10 (0.098)		
Craft/trades worker or entrepreneur × Prenatal education					0.89 (0.068)	0.88 (0.068)		
Intermediate (technical) occupation × No prenatal education					1.33^b^ (0.171)	1.33^b^ (0.170)		
Intermediate (technical) occupation × Prenatal education					0.82^c^ (0.089)	0.82^c^ (0.090)		
Managerial or higher intellectual occupation × No prenatal education					1.13^a^ (0.052)	1.13^a^ (0.051)		
Managerial or higher intellectual occupation × Prenatal education					0.78^a^ (0.045)	0.78^a^ (0.045)		
Crossed dummy variables for partner’s work status and prenatal education participation								
Unemployed × No prenatal education							1.20 (0.238)	1.20 (0.239)
Unemployed × Prenatal education							0.88 (0.079)	0.88 (0.079)
Not in labor force × No prenatal education							1.13 (0.099)	1.13 (0.100)
Not in labor force × Prenatal education							0.96 (0.117)	0.96 (0.118)
Working × No prenatal education							1.10^b^ (0.047)	1.10^b^ (0.048)
Working × Prenatal education							0.78^a^ (0.028)	0.78^a^ (0.028)
Epidemiologic and hospital controls	Yes	Yes	Yes	Yes	Yes	Yes	Yes	Yes
Other prenatal care and socioeconomic variables	Yes	Yes	Yes	Yes	Yes	Yes	Yes	Yes
Year fixed effects	Yes	Yes	Yes	Yes	Yes	Yes	Yes	Yes
Hospital effects	Fixed	Random	Fixed	Random	Fixed	Random	Fixed	Random
*N* (observations)	41,141	41,141	41,141	41,141	41,141	41,141	41,141	41,141

All regressions use the full sample of women and include a constraint: the sum of all the crossed variables equals 0. Epidemiologic control variables include woman’s demographics (age and parity) and medical risk factors (previous cesarean, diabetes, hypertension, eclampsia or preeclampsia, fetal growth restriction, placental bleeding, other obstetric pathology, plurality, term at delivery, fetal presentation, induced labor, and birth weight). Hospital control variables include hospital type (ownership status, equipment level, and teaching status), organization (day of delivery*,* obstetrician availability, and size), and staff (midwives, obstetricians, and anesthetists in FTEs per bed). Hospital invariant control variables (ownership status, equipment level, and teaching status) are only included in regressions with hospital random effects. Other prenatal care variables are trimester of the first antenatal visit, number of obstetric ultrasounds, nuchal translucency ultrasound, morphology ultrasound, and early prenatal interview. Other socioeconomic variables include woman’s familial situation, healthcare coverage, occupation, education except for columns 1–2 and work status except for columns 3–4, and her partner’s occupation except for columns 5–6 and work status except for columns 7–8. Robust standard errors in parentheses

^a^ = 1% significance level, ^b^ = 5%, ^c^ = 10%

## Abbreviations

*CNIL*: *Commission Nationale de l’Informatique et des Libertés*, French data protection commission authority
FTE: Full-time equivalent
*INSEE*: *Institut National de la Statistique et des Etudes Economiques*, French national institute of statistics and economic studies
*PCS*: *Premiers Certificats de Santé*, infant first health certificates
*SAE*: *Statistique Annuelle des Etablissements de santé*, French annual hospital statistics
US: United States
WHO: World Health Organization
